# X-ray dark-field radiography for in situ gout diagnosis by means of an ex vivo animal study

**DOI:** 10.1038/s41598-021-98151-0

**Published:** 2021-09-24

**Authors:** Josef Scholz, Nathalie Roiser, Eva-Maria Braig, Christian Petrich, Lorenz Birnbacher, Jana Andrejewski, Melanie A. Kimm, Andreas Sauter, Madleen Busse, Rüdiger Korbel, Julia Herzen, Daniela Pfeiffer

**Affiliations:** 1 Chair of Biomedical Physics, Department of Physics and Munich School of BioEngineering, Technical University of Munich, James-Franck Str. 1, 85748 Garching, Germany; 2 Clinic for Birds, Small Mammals, Reptiles and Omamental Fish, Centre for Clinical Veterinary Medicine, LMU Munich, 85764 Oberschleißheim, Germany; 3 Department of Diagnostic and Interventional Radiology, School of Medicine & Klinikum rechts der Isar, Technical University of Munich, 81675 Munich, Germany; 4 Department of Radiology, University Hospital, LMU Munich, 81377 Munich, Germany; 5 Institute for Advanced Study, Technical University of Munich, 85748 Garching, Germany

**Keywords:** X-rays, Imaging techniques, Gout, Radiography

## Abstract

Gout is the most common form of inflammatory arthritis, caused by the deposition of monosodium urate (MSU) crystals in peripheral joints and tissue. Detection of MSU crystals is essential for definitive diagnosis, however the gold standard is an invasive process which is rarely utilized. In fact, most patients are diagnosed or even misdiagnosed based on manifested clinical signs, as indicated by the unchanged premature mortality among gout patients over the past decade, although effective treatment is now available. An alternative, non-invasive approach for the detection of MSU crystals is X-ray dark-field radiography. In our work, we demonstrate that dark-field X-ray radiography can detect naturally developed gout in animals with high diagnostic sensitivity and specificity based on the in situ measurement of MSU crystals. With the results of this study as a potential basis for further research, we believe that X-ray dark-field radiography has the potential to substantially improve gout diagnostics.

## Introduction

Gout is a form of arthritis caused by the deposition of monosodium urate (MSU) crystals at peripheral joints^1^. The crystal deposition is caused by hyperuricemia defined by an elevated level of serum uric acid. Causes of hyperuricemia can be of primary or secondary nature including reduced tubular urate excretion^2^, endogenous overproduction of purines (tissue decay, chemotherapy, leukemia)^3^, or an excessive purine intake (purine rich diet, consumption of alcohol)^4,5^. Gout can lead to severe joint damage if left untreated and is often accompanied with cardiovascular, metabolic, or renal syndromes^6^. Therefore, a quick and reliable diagnosis is essential to prevent extending damage.

To date, the analysis of synovial fluid and the identification of MSU crystals using polarized light microscopy is the diagnostic standard of reference, however studies have shown that this method is rarely used in primary care facilities^7^. In addition, however, synovial fluid extraction is an invasive procedure that requires joint aspiration (arthrocentesis). Only chronic joint lesions caused by gout can be detected via conventional radiography, which is thus not suitable for the diagnosis of an acute gout arthritis^8^. Further imaging techniques such as ultrasound and dual-energy CT (DECT) have shown their potential in early diagnosis of gout and have been integrated into the new American College of Rheumatology (ACR) and European League Against Rheumatism (EULAR) classification criteria together with clinical characteristics^9^. Sonography is a time consuming examination which requires a well-trained examiner^10^. DECT is reported to have specificity rates of 75% to 100% in detecting MSU crystals^8^, but it is only available in specialized clinics so far and causes radiation exposure^10^.

Grating-based X-ray dark-field radiography is an advanced X-ray imaging technique that utilizes X-ray scattering to reveal microstructural information far below image resolution^11^. The origin of contrast of this imaging technique is small-angle scattering induced by micrometer-scale fluctuations in electron density. The potential of X-ray dark-field radiography has been investigated in different diagnostic fields over the last decade. Previous studies in animals have confirmed high diagnostic sensitivity of pulmonary emphysema and fibrosis, lung cancer, and pneumothorax^12–15^. Furthermore, human ex vivo studies have shown that dark-field radiography can be used to visualize otherwise non-resolvable microcalcifications and to assess the morphology of microcalcifications. In atherosclerosis, the presence of microcalcifications is considered an indicator of plaque stability, whereas the morphological characteristics provide information about the malignancy of the observed lesions in mammography^16–18^. A recent orientation study has demonstrated that X-ray dark-field radiography is able to detect MSU crystals in a murine gout model based on the injection of synthetic crystals^19^. To date, there is only sparse information available on the concentration of MSU at which inflammatory reactions are to be expected. Faires and McCarthy^20^ reported an amount of 6 mg MSU crystals above which inflammatory reactions were observed after injection into dog knees. Whether gout can be diagnosed by dark-field radiography is therefore difficult to answer by in vitro experiments.

In contrast to humans and primates, most other mammals metabolize purines to urea, which is then excreted in the urine^21^. The latter cannot develop gout naturally, so in classical animal experiments with mice, rats and rabbits, gouty arthritis needs to be artificially induced by injection of exogenous MSU crystals. To date, there are no environmentally or genetically induced animal models targeting the development of gouty arthritis^22^. Birds and reptiles on the other hand have a similar purine break down into uric acid and are therefore able to develop gout by MSU crystal deposition causing an inflammatory reaction corresponding to gouty arthritis^23^. This makes these animals a suitable model for in situ gout studies.

This work aims to evaluate the diagnostic value of X-ray dark-field radiography for the detection of MSU crystals in situ in animals with naturally developed gout by means of an explorative ex vivo animal study. In detail, we measured several different reptile joints with X-ray dark-field radiography and corresponding X-ray attenuation radiography, assessed the data in a reader study, and compared our results with polarized microscopy being the ground truth.

## Results

### Necropsy findings

**Figure 1:**
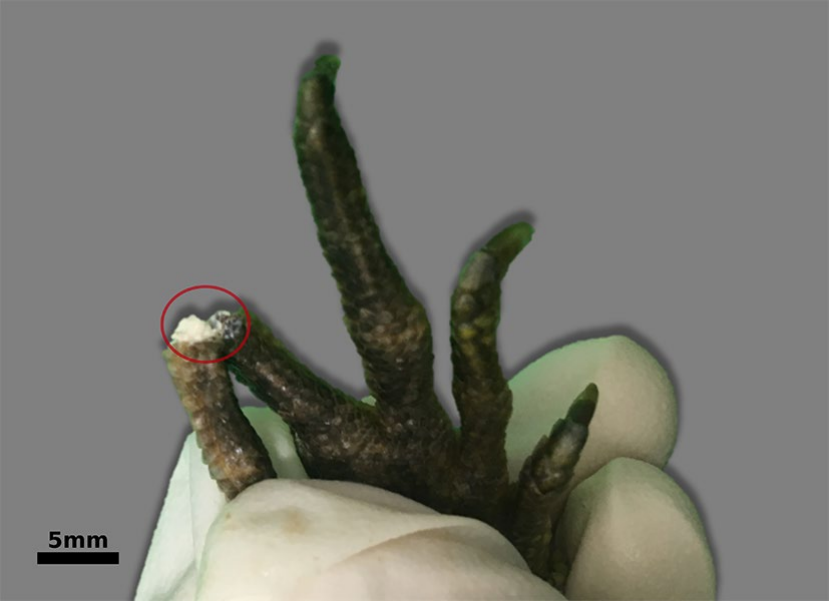
Hind limb of a water dragon with an opened metatarsophalangeal joint (red circle) containing chalky, white material.

Three of five animals had a history of suspected gout, which in two cases (one leopard gecko and one water dragon) could be confirmed by pathological examination and arthrocentesis. Both animals had severely deformed limbs. Especially the carpus, tarsus and the metatarsophalangeal joints were swollen and contained a chalky, white material. Figure 1 shows a hind limb of a water dragon with the described articular deposits in an opened proximal interphalangeal joint indicated by a red circle. In further pathological examination, the kidneys were swollen and lightened in color. The leopard gecko also showed multiple yellowish to white granulomas covering its serosa, especially the liver capsule.

### Arthrocentesis

Microscopically, MSU crystals were found in the joints of two animals. Table 1 indicates whether needle shaped MSU crystals were detected at all and the location of MSU crystals. The crystals were found to range in size from 2.5 to 56 µm, and the average size of the crystals varied between the different localizations (i.e. leopard gecko hip: 25 µm, tarsus: 8 µm, internal granuloma: 46 µm).

**Table 1:** Overview of the MSU crystal analysis findings and crystal deposition sites of all excised animal limbs.

Animal no.	Limb	MSU positive	MSU crystal location
Animal #1	Right hind	No	N/a
Animal #1	Left front	No	N/a
Animal #2	Right front	Yes	Elbow, carpus
Animal #2	Right hind	Yes	Metatarsophalangeal joint, tarsus, toe joints
Animal #3	Left front	No	N/a
Animal #4	Right front	No	N/a
Animal #5	Left front	Yes	Elbow, carpus
Animal #5	Left hind	Yes	Hip, metatarsophalangeal joint, knee

**Figure 2:**
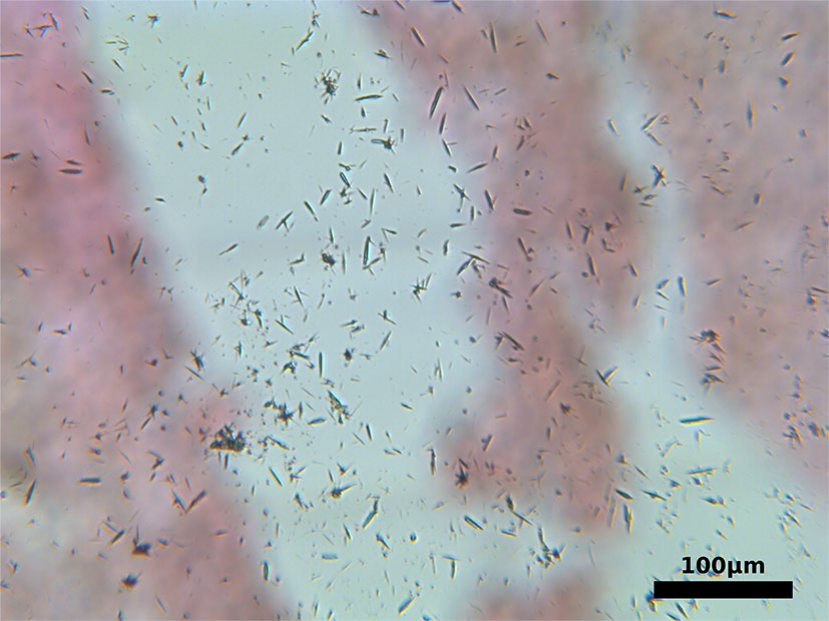
Microscope image of needle shaped MSU crystals, which were extracted from the knee of a leopard gecko.

Figure 2 shows a microscope image of needle shaped MSU crystals extracted from the knee of the leopard gecko.

### Dark-field measurements

**Figure 3:**
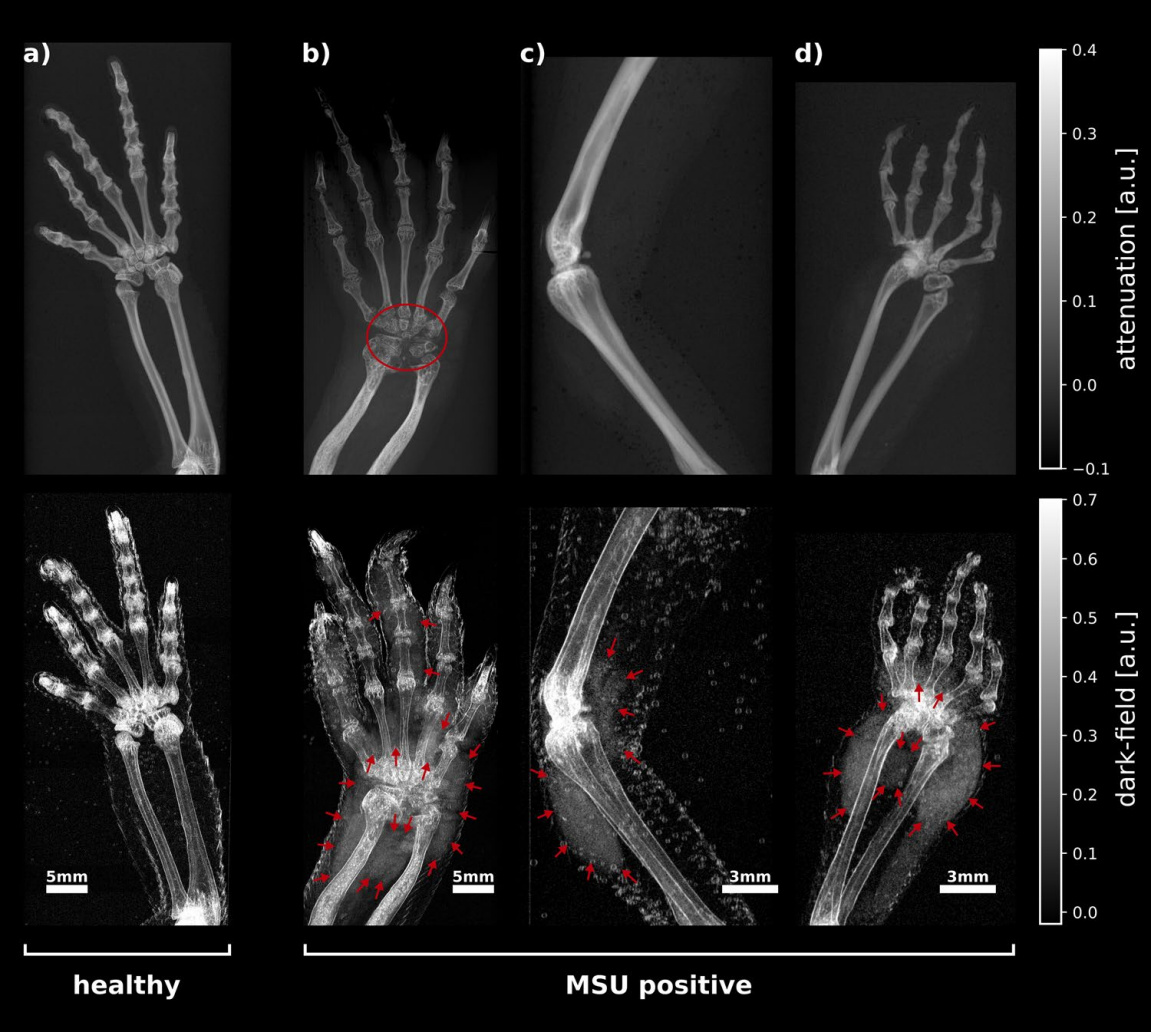
Attenuation (upper row) and dark-field (bottom row) radiographs of an MSU negative (‘healthy’) (**a**) and three MSU positive (**b**)–(**d**) reptile limbs. The attenuation images do not show any signs of MSU deposits. In (**b**) however, osseous destruction of the carpus are visible (red circle), which are a sign but do not explicitly indicate gout. In contrast to the healthy specimen, the X-ray dark-field radiographs of the MSU-positive samples show areas with increased signal in the peripheral soft tissue around joints and bones (red arrows), which indicate the presence of MSU crystals. The locations of these areal dark-field signals are consistent with regions in which MSU crystals were detected during pathological examinations. In all cases, the dark-field radiographs show a signal emanating from bones and joints and also from the water-soft tissue boundaries.

Prior to arthrocentesis, X-ray dark-field and attenuation radiographies of the excised animal limbs were taken. Figure 3 shows attenuation contrast (ATC) and dark-field contrast (DFC) radiographs of one healthy (Fig. 3a) and three proven MSU positive (Fig. 3b–d) excised animal limbs. In the attenuation radiography of the healthy sample a strong signal from bones can be measured, but no visible signs of bone deformation or crystal deposition. In the dark-field radiography there is also a signal from the bones and a stronger signal at the joints of the fingers as well as at the elbow. Further, a slight signal at the soft-tissue borders can be observed. The ATC radiographs of the MSU positive samples also show a signal originating from the bone. However, in contrast to the healthy specimen, bone deformations on the carpus in one of the MSU positive samples are visible (Fig. 3b, red circle). No ATC signal can be detected in the MSU positive samples indicating the presence of no MSU deposits. In contrast to the healthy sample, the dark-field radiographs of MSU positive samples show a strong signal in the soft tissue around joints and bones. In Fig. 3b–d the corresponding regions are indicated by red arrows. Figure 3b shows an extended dark-field signal in the soft tissue surrounding the carpus, metacarpals and finger joints. In the specimen shown in Fig. 3c, two areas with a strong dark-field signal are visible around the knee. Inside the third MSU positive sample (Fig. 3d), an increased dark-field signal can be observed in the soft tissue surrounding the carpus and the metacarpalia.

In all dark-field radiographs, a signal emanating from the bones is visible and more pronounced in the area of the joints. The visibility of bone in dark-field radiographs is attributable to the bone microstructure, but also partly to beam-hardening effects^24^. Accordingly, the beam-hardening induced dark-field signal correlates with the corresponding ATC radiographs. The increased signal from bone in areas close to joints originates from the microstructure in trabecular bone^25^. The slight dark-field signal occurring at soft tissue transitions is an artifact caused by strong electron density gradients^26,27^. Due to the same effect, small air bubbles adhering to the skin surface of the samples are also visible in the dark-field signal (cf. Fig. 3c). In contrast to these features, which occur in all dark-field images, extensive signals in peripheral soft tissue around joints occur only in the MSU positive samples and are not visible in the corresponding attenuation images. The locations of these areal dark-field signals are consistent with regions in which MSU crystals were detected during pathological examinations. The image impressions for the remaining samples were similar to the examples shown in Fig. 3. Radiographs of all animal samples as listed in Table 1 are available as supplementary digital content (Supplemental Digital Content 1, Figure SI 1 and Figure SI 2).

### Reader study

A reader study was conducted to evaluate the potential of dark-field imaging compared to standard absorption-based imaging for the detection of gout in situ.

The detection of MSU crystals was not possible based on attenuation radiographs alone (sensitivity = 0%, specificity = 87.5%). In contrast, evaluation based on dark-field data only (sensitivity = 60%, specificity = 100%) as well as the combination of dark-field and attenuation images (sensitivity = 60%, specificity = 100%) provided a much better diagnostic value. Each MSU-positive sample in this study had multiple regions of MSU deposits, which could not all be identified by the radiologists. However, at least one region was detected in each of the MSU-positive samples. For this purpose, if only the general presence of MSU crystals in the examined samples is taken into account, independent of a specification of certain regions, the sensitivity reaches a value of 100% with a specificity of 100% if dark-field images alone or dark-field in combination with attenuation data are provided.

Regarding the assessment of the dark-field data alone and the combined data of attenuation and dark-field data, there is a perfect agreement (κ = 1.000) between the individual readers in both cases. The calculation of a Cohen’s Kappa was not possible in the case of attenuation images only because the assessment of one reader showed zero variance (no sample was given a positive diagnosis of gout by this reader).

**Figure 4:**
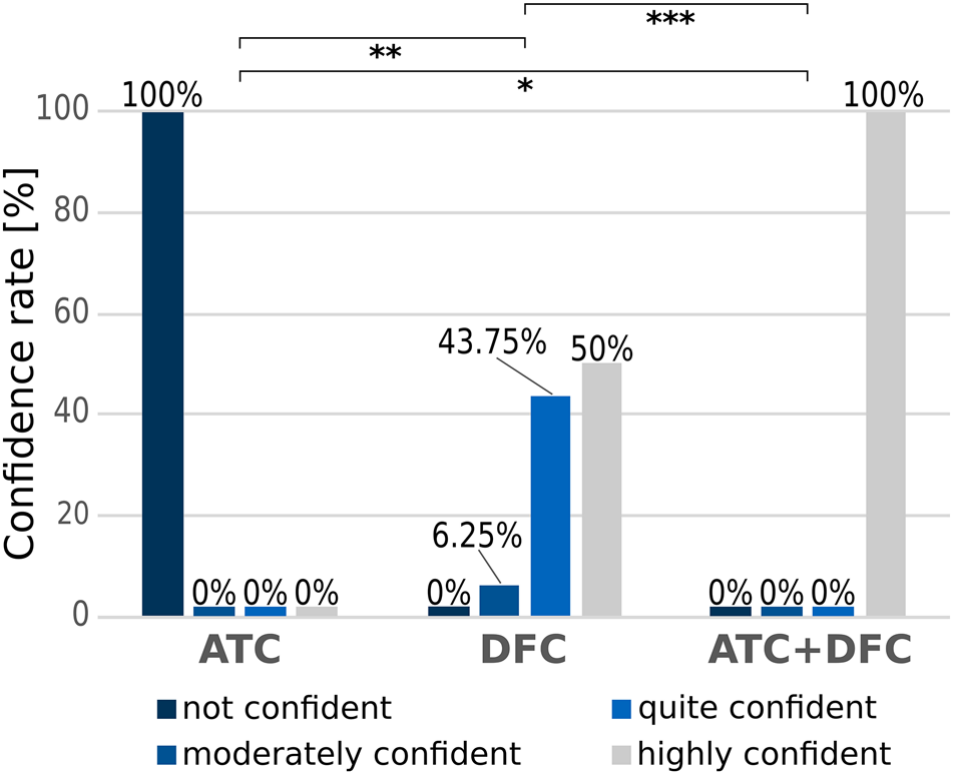
Summary of the confidence ratings given by the readers based on attenuation only (ATC), dark-field only (DFC) and the combination of attenuation and dark-field data (ATC + DFC). There was a significant difference in the confidence scores between the attenuation only and dark-field only (**p = 0.001) as well as the attenuation only and the combined attenuation and dark-field data (*p = 0.001). Between the dark-field only and the combined attenuation and dark-field data, there was no significant difference (***p = 0.157).

Figure 4 shows the distribution of confidence scores for all three scenarios, where either only attenuation images, only dark-field images, or both attenuation and dark-field images are available simultaneously. In the assessment of attenuation images alone the readers reported a confidence level of 1 (‘not confident’) in 100% of cases, in the assessment of dark-field images alone a confidence level of 3 (‘quite confident’) in 43.75% and 4 (‘highly confident’) in 50% of all cases. For the evaluation of the combined attenuation and dark-field data, the confidence level was 4 (‘highly confident’) in 100% of all cases. The confidence ratings differed significantly between attenuation only and the combined dark-field and attenuation data assessment (z-score = − 4.950, p_corr_ ≤ 0.001) and the attenuation only and the dark-field only data assessment (z-score = − 3.536, p_corr_ = 0.001). No significant difference was observed between the dark-field only and the combined attenuation and dark-field data assessment (z-score = − 1.414, p_corr_ = 0.157).

## Discussion

**Figure 5:**
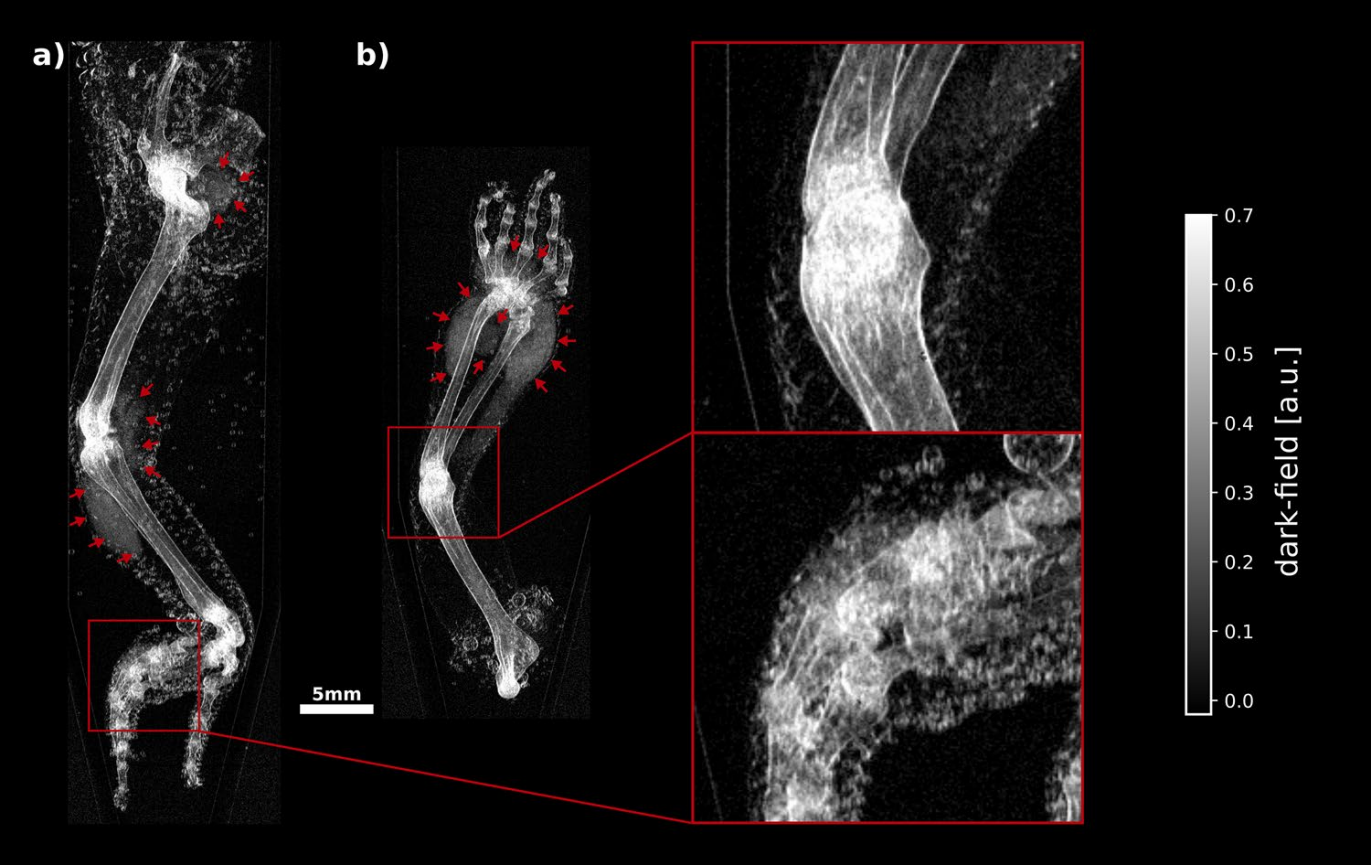
Exemplary darkfield image of two sample in which not all MSU deposits could be detected by the radiologists. The hind limb of animal #5 (**a**) contained MSU deposits in three different regions: Hip, knee (indicated by red arrows) and the metatarsophalangeal joints (red box). All regions show a more or less increased dark-field signal. From the zoom-in box it is clear that the detection of such areas can be made significantly more difficult by the increased presence of adhering air bubbles. The front limb (**b**) of the same animal contained MSU deposits around the carpus (indicated by red arrows) and the elbow (red box). As in the area of the carpus, a widely extending dark field signal can be seen, this cannot be observed in the area of the elbow.

In total, MSU crystals distributed over ten separate regions could be identified in four of the eight excised limbs via crystal analysis using light microscopy, and the presence of a dark-field signal could be visually observed in MSU-positive regions. The reader study showed that based on attenuation images alone, the detection of MSU crystals is not possible (sensitivity = 0%). Although the attenuation image in Fig. 3b already displays bone deformities, this is not explicitly a sign of gout and therefore was not labeled as gout positive by the readers. Based on the dark-field and combined dark-field and attenuation data, overall MSU positivity within the excised limbs could be diagnosed with a specificity and sensitivity of 100%. However, several samples contained multiple MSU positive regions, of which not all could be identified by the readers. The sensitivity under consideration of individual crystal deposits was 60% for the dark-field only as well as the combined dark-field and attenuation data. This can be caused by a variety of factors, such as the dependence of the strength of the dark field signal on the structure size and concentration of the crystals^28,29^, as well as the properties of the measured samples per se. A correlation between the structural size of the extracted crystals and false negatives was not observed within this study. Since the crystals were not artificially injected and the extracted crystals do not allow conclusions to be drawn about the concentrations in the tissue and synovial fluid, correlation with any concentrations was not possible. Dark-field signals originating from the squamulose skin structure or adhered air bubbles can further complicate the detection of MSU crystals in squamata. The necessity to measure the samples submerged in water inside Falcon™ tubes and the rigid condition of the ex-vivo samples have further complicated an accurate positioning of the samples, so that in some cases overlapping of individual fingers in the corresponding radiographs could not be avoided. For example, the hind limb of animal #5 (Fig. 5a) contained MSU deposits at the hip, knee, and metatarsophalangeal joints, but of these, the metatarsophalangeal joints could not be identified as MSU-positive by the radiologists. All corresponding regions show extended dark-field signals, indicated by red arrows and, in the case of the metatarsophalangeal joints, by a red box. It is noticeable that a particularly large number of air bubbles have accumulated in the area of the metatarsophalangeal joints, which could presumably have made it more difficult for the radiologists to detect MSU crystals in this area. Another possible reason for the reduced sensitivity may be the difficulty of detecting small amounts of MSU crystals that are confined to the intra-articular space. Figure 5b shows a forelimb specimen from animal #5. In this specimen MSU crystals could be extracted from the carpus as well as the elbow. As in the area of the carpus, a broad dark-field signal s visible, this cannot be observed in the area of the elbow. MSU crystals located exclusively in the synovial fluid cannot be detected with the experimental setup of this study because the image resolution is not sufficient to resolve the intra-articular space of the animals used. Although the aforementioned limitations were readily observed in the dark-field radiographs, the influence of low crystal concentrations on the detectability of MSU deposits still cannot be excluded.

In terms of confidence ratings, confidence was higher based on the combined dark-field and attenuation data than on the dark-field-only data, although there was no difference in sensitivity and specificity. By incorporating attenuation radiographs, dark-field contrast signals that do not arise explicitly from scattering can be identified. Hence, MSU deposits can be recognized more reliably.

Besides gouty arthritis, there are other crystal-related arthropathies caused by the deposition of calcium pyrophosphate dihydrate (CPPD) or basic calcium phosphate (BCP). So far, there are no dark-field studies on CPPD and BCP crystals. However, CPPD and BCP deposits are detectable and can even be distinguished from each other by conventional radiography^30^. Because of the multimodality of dark-field imaging, it should be possible to rule out confusion with other crystal-related arthropathies on the basis of the attenuation contrast images that are simultaneously available.

Due to the intent of this explorative study to evaluate the diagnostic value of dark-field imaging for the detection of gout in situ by means of animal samples with naturally developed gout, the scope of the statistics is limited by the availability of animal specimens with suspected gout. The occurrence of gout among animals is mainly restricted to birds and reptiles. Further, pathological examination revealed that in the animals with proven gout show that the disease was already well progressed. The extraction of white chalky material indicates high MSU crystal concentrations in the measured limbs. The question whether dark-field imaging can be used to detect early gout cannot be answered within this work.

To date, the standard of reference for a definite diagnosis of gout is the synovial fluid analysis using polarized light microscopy^31,32^. Although the identification of MSU crystals is crucial for a definite diagnosis of gout, synovial fluid analysis is rarely used in clinical routine^33^. The extraction of synovial fluid is an invaluable but invasive procedure, which can be associated with risk of infection and discomfort depending on the patient^34^. Sensitivity and specificity of synovial fluid analysis are furthermore strongly dependent on the training level of the respective observer^35^. Various studies have demonstrated poor consistency among different observers in the identification of MSU crystals using polarized light microscopy^35,36^. In addition, non-invasive imaging methods with equally high or higher specificity exist for gout diagnostics. Ultrasound (US) is a widely used imaging technique in clinical routine and has a high specificity for crystal depositions in the form of tophi, on the articular surface of hyaline cartilage and within the joint space. However, ultrasound has a modest to low sensitivity in the diagnosis of gout and the performance is strongly influenced by the equipment used and the experience of the sonographer^37,38^. Plain X-ray radiography is suitable for supporting diagnosis in progressed or late stages of gout which are associated with irreversible joint damage but has only limited value for the detection of MSU crystals^39^. A more advanced X-ray imaging technique is DECT. DECT offers high sensitivity and specificity and also allows the detection of extra-articular gout, which otherwise may result in a false negative after joint aspiration^40^.

X-ray dark-field radiography is a non-invasive imaging technique that offers high sensitivity and—unlike existing methods such as the synovial fluid analysis or ultrasound—a high specificity for the detection of MSU crystals. In addition, X-ray dark-field imaging is a multimodal imaging technique, which simultaneously provides conventional X-ray attenuation data^41^. This additional source of contrast can thus be used to visualize further features of progressed gout such as bone erosion or joint narrowing^8^, as well as potentially distinguish MSU from CPPD and BCP deposits^30^.

Medical applications of dark-field imaging have been intensively investigated, but the technique is not yet used in clinical routine. Initial clinical prototypes for lung imaging^42^ and mammography^43,44^ already have been developed, and some are already in pilot operation as part of clinical trials^45–47^. One of these studies is focused on cartilage imaging for the diagnosis of rheumatoid arthritis^45^. The imaging device described here was developed for imaging human hands and is hence potentially suitable for the diagnosis of gouty arthritis. The dark-field images from the first patient study with this device show that the intra articular space can be resolved in human joints and artifacts emanating from the skin surface are not to be observed^46^. This suggests that, with regard to human application, it will not be necessary to immerse joints in fluid. However, it cannot be excluded that effects originating from the skin surface may still occur with a dark-field setup sensitive enough to detect MSU crystals. This can only be answered conclusively by future studies. In the context of radiation exposure, it has already been demonstrated that dark-field radiography is feasible even at dose levels below low-dose CT^47^.

Certainly, prior to clinical studies, pending issues such as dose, technical requirements of a dark-field imaging setup dedicated to the diagnosis of gouty arthritis, as well as a lower detection limit still need to be addressed within future studies. This involves determining what minimum requirements a Talbot-Lau interferometer must meet in order to be as sensitive as possible to MSU crystals as well as determining a lower detection limit for low MSU concentrations, which is essential to establish comparability with other methods for gout diagnosis. Furthermore, especially with regard to clinical applications, it is necessary to assess the diagnostic value of X-ray dark-field radiography for the detection of MSU crystals in the context of clinical doses. To address these issues in advance of any clinical trials, a study design should be chosen that provides a higher degree of control and statistics compared with our study. Studies based on the injection of exogenous MSU crystals seem to be the most suitable option for this purpose.

In summary, we have demonstrated that X-ray dark-field imaging can visualize MSU crystal deposits and offers a high diagnostic value for the detection of gout in situ in animals with naturally developed gout. The results of this proof-of-concept study represent the first demonstration that gouty arthritis can be detected in situ using dark-field radiography. Certainly, future studies dedicated to the detectability of MSU crystals within a clinical context are necessary. Based on our results and the fact that dark-field radiography is already feasible in a clinical context we believe that X-ray dark-field radiography has the potential to substantially improve gout diagnostics.

## Materials and methods

### Grating-based X-ray dark-field imaging

**Figure 6:**
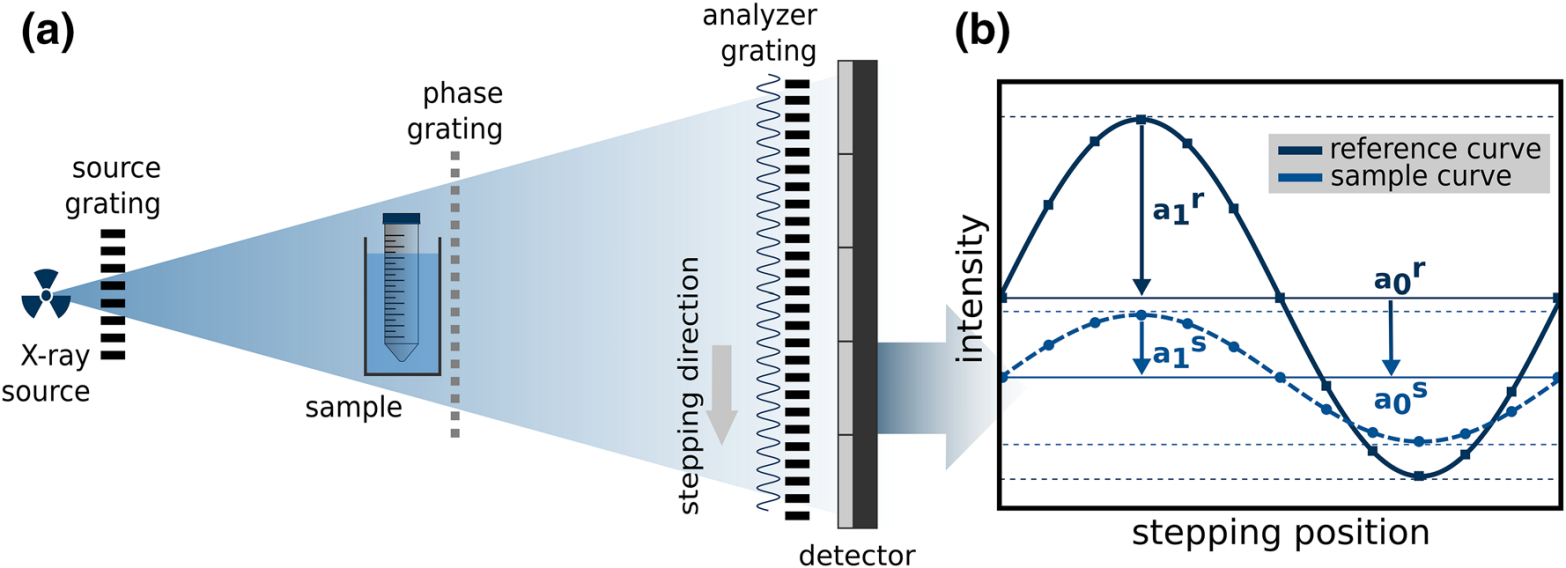
Schematic overview of the experimental X-ray dark-field setup (**a**) and the signal extraction mechanism (**b**). Downstream of the X-ray source the individual gratings (source-, phase-, and analyzer-grating) are arranged. The sample is positioned in front of the phase-grating. The source grating is used to split an extended source spot into mutually coherent line sources to provide sufficient beam coherence. The interference pattern, induced by the phase grating, is sampled via a stepwise lateral movement of the analyzer grating. From the resulting stepping curves (**b**) each pixel attenuation and dark-field contrast data can be extracted. The attenuation of X-ray results in a mean intensity (a_0_) decrease of this curve. Scattered X-rays reduce the interference pattern visibility, which is related to a mean amplitude (a_1_) decrease and the origin of the dark-field contrast.

Grating-based X-ray dark-field imaging is an interference-based imaging technique, which utilizes X-ray small angle scattering arising from subresolutional refractive index fluctuations to visualize otherwise inaccessible structural information beyond the actual image resolution^41^. For conventional laboratory setups with low brilliance X-ray sources, the use of a Talbot-Lau interferometer (TLI) is essential for this technique. In general, a total of three X-ray gratings are necessary to form a TLI. Figure 6a shows an example of the configuration of such a grating-based X-ray setup. As a central component of such an interferometer, a phase-grating is employed to generate a periodic interference pattern. This interference pattern can usually not directly be resolved by conventional X-ray detectors, so an additional absorption grating (analyzer grating) is used for sampling the interference pattern. A second absorption grating (source grating) is used to create an array of individually coherent but mutually incoherent line sources from an extended X-ray source, since the spatial coherence requirements necessary for interference effects are not met with conventional laboratory X-ray sources^48^.

The stepwise lateral movement of one of the three gratings over one grating period yields an image series, which is combined to obtain a sinusoidal intensity curve for each detector pixel (cf. Figure 6b). This technique is referred to as phase-stepping, more detailed information can be found in^49^. From this intensity curve the parameters necessary for attenuation and dark-field contrast can be extracted simultaneously. The origin of the dark-field signal is an X-ray scattering induced degradation of the interference pattern, which leads to an amplitude reduction of the sinusoidal stepping curve. The attenuation signal is simultaneously accessible via the mean value of the stepping curve. To measure the effects on the interference pattern induced by a sample, an additional reference scan without sample must be acquired. From the stepping curve parameters of the sample and the reference scan, the dark-field contrast (DFC) and attenuation contrast (ATC) can be calculated as follows:

$$DFC=1-\frac{{a}_{1}^{s}{a}_{0}^{r}}{{a}_{1}^{r}{a}_{0}^{s}}$$

$$ATC=1- \frac{{a}_{0}^{s}}{{a}_{0}^{r}}$$

where ${a}_{1}^{s}, {a}_{1}^{r}$ denote the amplitudes and ${a}_{0}^{s}, {a}_{0}^{r}$ denote the mean values of the sample and the reference scan, respectively.

### Experimental Talbot-Lau setup

The X-ray source used in the experimental setup is a RA-Micro7 HFMR tabletop rotating anode X-ray generator (Rigaku Inc., USA) equipped with a molybdenum target. The Talbot-Lau interferometer is implemented in a symmetrical configuration with an overall length of 1.7 m. The structuring elements of the gratings are made of gold with a period of 5.4 µm. The phase grating produces a phase-shift of π for the design energy of 27 keV with a structure height of 5.2 µm. The source- and the analyzer grating have a structure height of 65 µm and 70 µm respectively. The X-ray camera used in this experimental setup is a SANTIS photon-counting detector (DECTRIS Ltd., Baden, Switzerland) with a 750 µm thick cadmium telluride (CdTe) sensor and a pixel size of 75 µm. For one measurement a gallium arsenide (GaAs) sensor with 450 µm thickness was used^50^.

### Animals

All animals examined in this study were collected from a veterinary clinic and not specifically bred in a laboratory. Since the collected animals already perished in a natural way or were put to sleep for animal protection reasons, this is by definition not an animal experiment requiring ethical approval. By means of a study call freshly deceased reptiles with suspected gout or chronic kidney disease were collected. For each animal a negative control animal of the same species was looked for. Simultaneously animals of the study facility (companion and wild animals) showing the fitting phenotype were examined and integrated into the study. A total of five animals from the zoological group of Squamata were examined within this study: Three bearded dragons (*Pogona vitticeps*), one water dragon (*Physignathus cocincinus*), and one leopard gecko (*Eublepharis macularius*).

**Figure 7:**
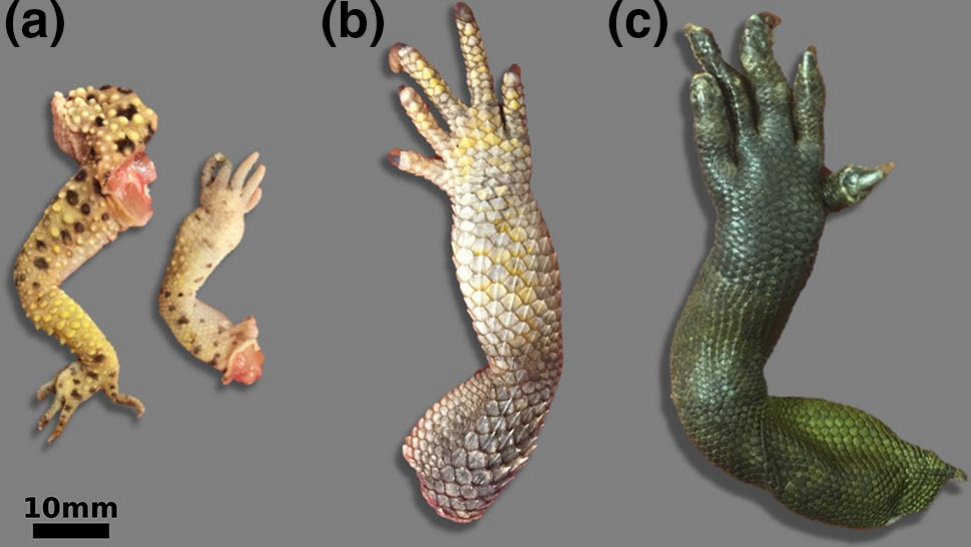
Exemplary selection of the animal limbs examined within this study. Excised limbs of a leopard gecko (**a**), a bearded dragon (**b**), and a water dragon (**c**).

Each animal was examined pathologically following an established protocol. Firstly, the animal was identified and labelled. Particular attention was paid to abnormalities in the joints. The animal body was opened, and all organs were assessed according to their size, shape and color. Particular attention was paid to abnormalities of the abdominal serosae, the pericardium, the liver capsule, and kidneys. After the examination, the limbs were separated and stored at – 20 °C until further use. The excised limbs of one leopard gecko, one bearded dragon and one water dragon are shown in Fig. 7. Fixation in formalin was not possible as uric acid crystals dissolve from the tissue in aqueous solutions. A total of n = 8 limbs was used for the present animal study.

### X-ray dark-field measurement parameters

During the radiography measurement the respective samples were immersed in water inside Falcon™ tubes (Thermo Fisher Scientific Inc., Waltham, USA) of 17 to 30 mm diameter for only a short time to prevent X-ray scattering effects arising from the tissue surface. Additionally, the Falcon™ tubes also were immersed in water using PMMA containers to minimize the influence of spectral effects. Depending on the size of the used Falcon™ tube, water containers with thicknesses of 2 cm or 4 cm were used. Note that the reference scans were also conducted with the respective water container in the beam path.

For the measurements the samples were placed directly in front of the phase grating. This geometrical arrangement results in an effective pixel size of (36 × 36) µm^2^ for the acquired radiographs. During the measurements the rotating anode X-ray source was operated at a voltage of 50 kVp and a tube current of 24 mA. The individual sample measurements were conducted by moving the source grating over one grating period in eleven equidistant steps. The exposure time for each step was set to 10 s (30 s in one single case). The reference scans were recorded with the threefold exposure time compared to the sample scan. For the processing of the stepping data, an expectation maximization algorithm as described in^51^ was applied.

### Arthrocentesis

Following the measurements in the dark field scanner, adapted arthrocentesis was performed. In order to obtain as much sample material as possible the joints were opened with a section knife. Imprints were made from the opened joints and the surrounding soft tissue and examined under the microscope (Aristoplan, Leica, Germany) by a veterinarian for the presence of needle-shaped crystals. Images were taken with a camera (Leica MC 190 HD, Leica, Germany) and edited with the Leica Application Suite program.

### Reader study

A reader study was conducted to evaluate the potential benefit of X-ray dark-field imaging for the detection of gout in situ. For the study, attenuation and dark-field images of eight limbs from the five animals were presented to two radiologists with seven and more than 10 years of experience. Four of the eight cases contained proven MSU crystals (positive samples), the remaining four cases did not contain proven MSU crystals (negative control). The study was divided into three separate sessions. In the first session the radiologists were shown only attenuation and in the second session only dark-field contrast images. In the third session the radiologists were simultaneously provided with attenuation and dark-field contrast data. The three consecutive sessions were performed with a time delay and randomized image order to minimize recall bias. One healthy animal (bearded dragon) was excluded from the actual reader study and was used as a training example for the readers. Prior to the first reader session, readers were given a brief overview of the measurement procedure and a short introduction to dark-field imaging using a healthy test sample not included in the reader study and a sample consisting of isolated MSU crystals in water. Although the readers already have experience with dark-field images as they have participated in previous studies, they received an introduction into reading dark-field images. For each session, the radiologists were asked to indicate if they could detect the presence of MSU crystals and if so, to specify the corresponding location. They were further asked to indicate how confident they were in their assessment of each case using a 4-point scale ranging from 1 to 4 (1 = ‘not confident’, 2 = ‘moderately confident’, 3 = ‘quite confident’, 4 = ‘highly confident’).

### Statistical analysis

To assess the performance of the individual imaging modalities, sensitivity and specificity were used as statistical measures. The inter-rater reliability between the individual readers was quantified using Cohen's Kappa (κ). To analyze how significantly the confidence levels of the readers differ between the individual imaging modalities, a non-parametric Friedman test for related samples was conducted and a Bonferroni correction was calculated for the pairwise comparisons (IBM SPSS Statistics for Windows, Version 27.0, IBM Corp, USA). The significance level for this comparison was set at p = 0.05.

## Supplementary Information

Supplementary Information.

## Data Availability

The data that support the findings of this study are available from the corresponding author upon reasonable request.

## References

[1] Porter R, Rousseau GSI (2000). Gout: The Patrician Malady.

[2] Pascart T, Lioté F (2018). Gout: State of the art after a decade of developments. Rheumatology.

[3] Schöffel D (2013). Gicht ist mehr als nur Podagra. Orthopädie Rheuma.

[4] Choi HK, Atkinson K, Karlson EW, Willett W, Curhan G (2004). Purine-rich foods, dairy and protein intake, and the risk of gout in men. N. Engl. J. Med..

[5] Choi HK, Atkinson K, Karlson EW, Willett W, Curhan G (2004). Alcohol intake and risk of incident gout in men: A prospective study. Lancet.

[6] Kuo C-F, Grainge MJ, Zhang W, Doherty M (2015). Global epidemiology of gout: Prevalence, incidence and risk factors. Nat. Rev. Rheumatol..

[7] Owens D, Whelan B, McCarthy G (2008). A survey of the management of gout in primary care. Irish Med. J..

[8] Omoumi P, Zufferey P, Malghem J, So A (2016). Imaging in gout and other crystal-related arthropathies. Rheum. Dis. Clin..

[9] Neogi T (2015). 2015 gout classification criteria: An American College of Rheumatology/European League Against Rheumatism collaborative initiative. Arthritis Rheumatol..

[10] Ragab G, Elshahaly M, Bardin T (2017). Gout: An old disease in new perspective—A review. J. Adv. Res..

[11] Malecki A, Potdevin G, Pfeiffer F (2012). Quantitative wave-optical numerical analysis of the dark-field signal in grating-based X-ray interferometry. EPL Europhys. Lett..

[12] Schleede S (2012). Emphysema diagnosis using X-ray dark-field imaging at a laser-driven compact synchrotron light source. Proc. Natl. Acad. Sci. USA.

[13] Yaroshenko A (2015). Improved in vivo assessment of pulmonary fibrosis in mice using X-ray dark-field radiography. Sci. Rep..

[14] Scherer K (2017). X-ray dark-field radiography—In-vivo diagnosis of lung cancer in mice. Sci. Rep..

[15] Hellbach K (2018). Depiction of pneumothoraces in a large animal model using x-ray dark-field radiography. Sci. Rep..

[16] Hetterich H (2017). Dark-field imaging in coronary atherosclerosis. Eur. J. Radiol..

[17] Grandl S (2015). Improved visualization of breast cancer features in multifocal carcinoma using phase-contrast and dark-field mammography: An ex vivo study. Eur. Radiol..

[18] Scherer K (2016). Improved diagnostics by assessing the micromorphology of breast calcifications via X-ray dark-field radiography. Sci. Rep..

[19] Braig em (2016). X-ray Dark-Field Radiography: Potential for Visualization of Monosodium Urate Deposition. Investig. Radiol..

[20] Faires J, McCarty D (1962). Acute arthritis in man and dog after intrasynovial injection of sodium urate crystals. Lancet.

[21] Nelson D, Cox M (2011). Lehninger Biochemie.

[22] Lu J (2019). Mouse models for human hyperuricaemia: A critical review. Nat. Rev. Rheumatol..

[23] Carretero A, König HE, Liebich H-G, Hinterseher C, Korbel R, König HE, Korbel R, Liebich H-G (2009). Harnorgane (Organa urinaria). Anatomie der Vögel.

[24] Yashiro W, Vagovic P, Momose A (2015). Effect of beam hardening on a visibility-contrast image obtained by X-ray grating interferometry. Opt. Express.

[25] Jud C (2017). Trabecular bone anisotropy imaging with a compact laser-undulator synchrotron x-ray source. Sci. Rep..

[26] Yang Y, Tang X (2012). The second-order differential phase contrast and its retrieval for imaging with x-ray Talbot interferometry. Med. Phys..

[27] Yashiro W, Momose A (2015). Effects of unresolvable edges in grating-based X-ray differential phase imaging. Opt. Express.

[28] Bech M (2012). Experimental validation of image contrast correlation between ultra-small-angle X-ray scattering and grating-based dark-field imaging using a laser-driven compact X-ray source. Photon. Lasers Med..

[29] Prade F, Yaroshenko A, Herzen J, Pfeiffer F (2015). Short-range order in mesoscale systems probed by X-ray grating interferometry. EPL Europhys. Lett..

[30] Omoumi P, Zufferey P, Malghem J, So A (2016). Imaging in gout and other crystal-related arthropathies. Rheum. Dis. Clin. N. Am..

[31] Pascual E, Sivera F, Andres M (2011). Synovial fluid analysis for crystals. Curr. Opin. Rheumatol..

[32] Malik A, Schumacher HR, Dinnella JE, Clayburne GM (2009). Clinical diagnostic criteria for gout: Comparison with the gold standard of synovial fluid crystal analysis. J. Clin. Rheumatol..

[33] Petersel D, Schlesinger N (2007). Treatment of acute gout in hospitalized patients. J. Rheumatol..

[34] Courtney P, Doherty M (2013). Joint aspiration and injection and synovial fluid analysis. Best Pract. Res. Clin. Rheumatol..

[35] Lumbreras B (2005). Analysis for crystals in synovial fluid: Training of the analysts results in high consistency. Ann. Rheum. Dis..

[36] Graf SW, Buchbinder R, Zochling J, Whittle SL (2013). The accuracy of methods for urate crystal detection in synovial fluid and the effect of sample handling: A systematic review. Clin. Rheumatol..

[37] Lee YH, Song GG (2018). Diagnostic accuracy of ultrasound in patients with gout: A meta-analysis. Semin. Arthritis Rheum..

[38] Zhang Q (2018). The diagnostic performance of musculoskeletal ultrasound in gout: A systematic review and meta-analysis. PLoS ONE.

[39] Richette P (2020). 2018 updated European League Against Rheumatism evidence-based recommendations for the diagnosis of gout. Ann. Rheum. Dis..

[40] Chou H, Chin TY, Peh WC (2017). Dual-energy CT in gout—A review of current concepts and applications. J. Med. Radiat. Sci..

[41] Pfeiffer F (2008). Hard-X-ray dark-field imaging using a grating interferometer. Nat. Mater..

[42] Willer K (2018). X-ray dark-field imaging of the human lung-A feasibility study on a deceased body. PLoS ONE.

[43] Koehler T (2015). Slit-scanning differential x-ray phase-contrast mammography: Proof-of-concept experimental studies. Med. Phys..

[44] Arboleda C (2020). Towards clinical grating-interferometry mammography. Eur. Radiol..

[45] Momose A (2014). X-ray phase imaging: From synchrotron to hospital. Philos. Trans. R. Soc. A Math. Phys. Eng. Sci..

[46] Yoshioka H (2020). Imaging evaluation of the cartilage in rheumatoid arthritis patients with an x-ray phase imaging apparatus based on Talbot-Lau interferometry. Sci. Rep..

[47] Willer, K.*, et al.* X-ray dark-field chest imaging can detect and quantify emphysema in COPD patients. *SSRN Electron. J.* (2021).

[48] Pfeiffer F, Weitkamp T, Bunk O, David C (2006). Phase retrieval and differential phase-contrast imaging with low-brilliance X-ray sources. Nat. Phys..

[49] Weitkamp T (2005). X-ray phase imaging with a grating interferometer. Opt. Express.

[50] Scholz J (2020). Biomedical x-ray imaging with a GaAs photon-counting detector: A comparative study. APL Photonics.

[51] Marschner M (2016). Helical X-ray phase-contrast computed tomography without phase stepping. Sci. Rep..

