# Supplementary material for: X-ray dark-field radiography for in situ gout diagnosis by means of an ex vivo animal study
Source: Sci Rep. 2021 Sep 24;11:19021. doi: 10.1038/s41598-021-98151-0 (PMC8463704; doi:10.1038/s41598-021-98151-0)
Supplement: Supplementary file 1 — Supplementary Information. [file 41598_2021_98151_MOESM1_ESM.docx]

**Supplemental Digital Content 1**


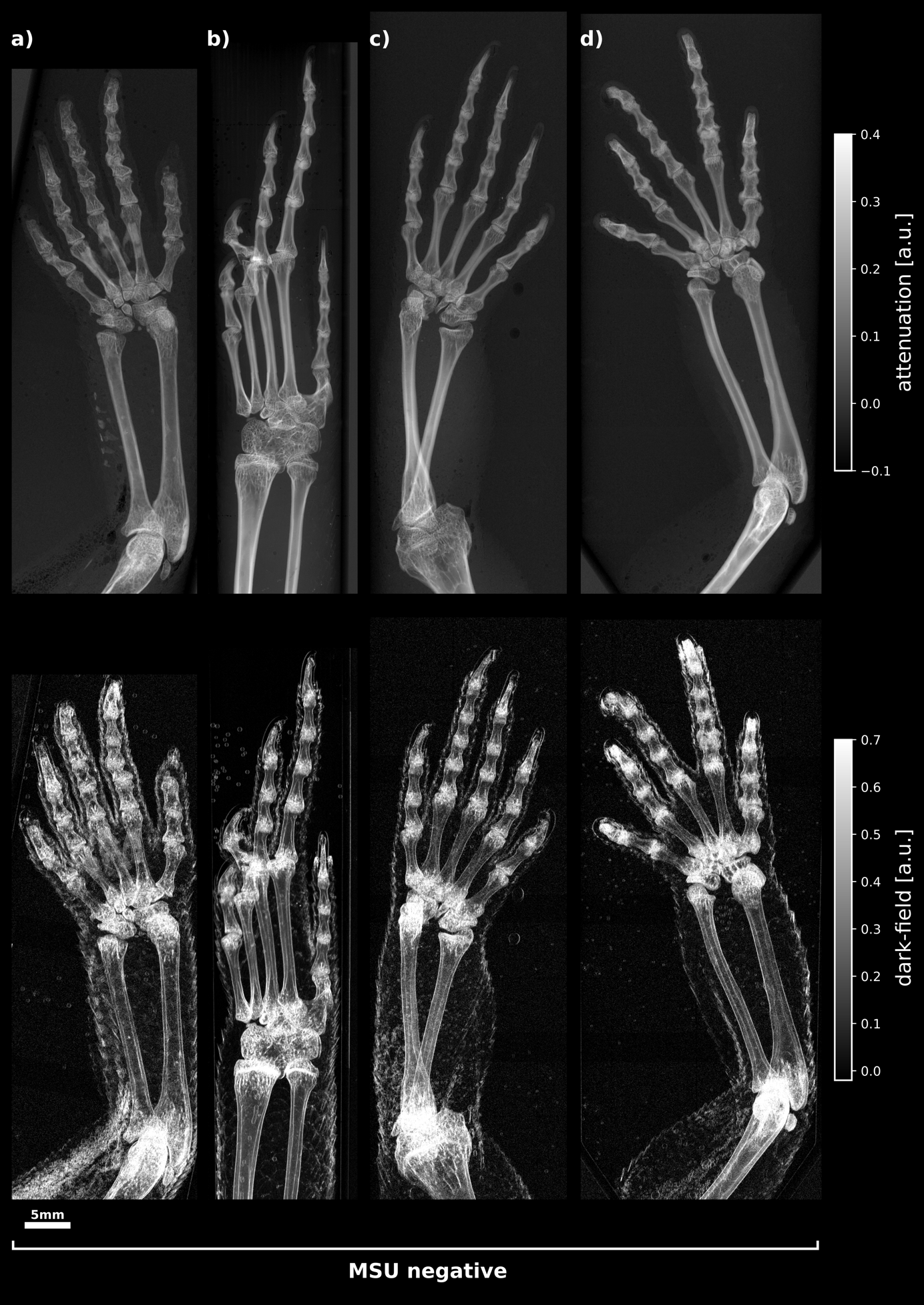


**Figure SI 1.** Attenuation (upper row) and dark-field (bottom row) radiographs of four MSU negative reptile limbs: a) animal #4, right front, b) animal #1, right hind, c) animal #1, left front and d) animal #3, left front. Neither the attenuation nor the dark-field images show any signs of MSU deposits.


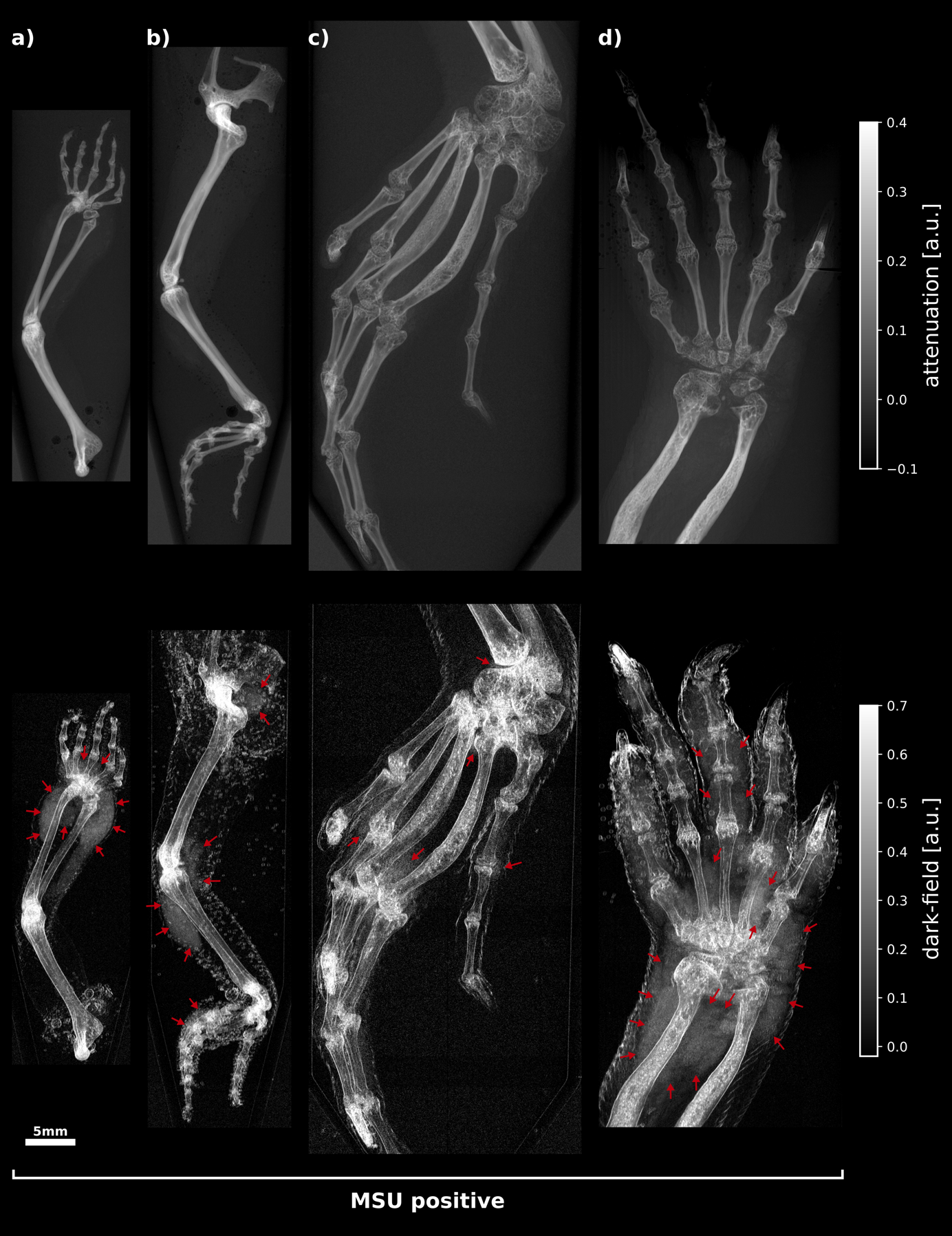


**Figure SI 2.** Attenuation (upper row) and dark-field (bottom row) radiographs of four MSU positive reptile limbs: a) animal #5, left front, b) animal #5, left hind, c) animal #2, right hind and d) animal #2 right front. The attenuation images do not show any signs of MSU deposits. In contrast to the healthy specimen, the X-ray dark-field radiographs of the MSU-positive samples show areas with increased signal in the peripheral soft tissue around joints and bones (red arrows), which indicate the presence of MSU crystals. The locations of these areal dark-field signals are consistent with regions in which MSU crystals were detected during pathological examinations. In all cases, the dark-field radiographs show a signal emanating from bones and joints and also from the water-soft tissue boundaries.
